# Outcomes in intervention research on snakebite envenomation: a systematic review

**DOI:** 10.12688/f1000research.122116.1

**Published:** 2022-06-08

**Authors:** Soumyadeep Bhaumik, Deepti Beri, Jyoti Tyagi, Mike Clarke, Sanjib Kumar Sharma, Paula R Williamson, Jagnoor Jagnoor

**Affiliations:** 1 Injury Division, The George Institute for Global Health, Faculty of Medicine, University of New South Wales,, Sydney, New South Wales, 2042, Australia; 2 Injury Division, The George Institute for Global Health, New Delhi, Delhi, 110025, India; 3 Meta-research and Evidence Synthesis Unit, George Institute for Global Health, New Delhi, Delhi, 110025, India; 4 Centre for Public Health, School of Medicine, Dentistry and Biomedical Sciences, Queen's University Belfast, Belfast, UK; 5 Department of Internal Medicine, B.P. Koirala Institute of Health Sciences, Dharan, Nepal; 6 Department of Health Data Science, Institute of Population Health, University of Liverpool, Liverpool, UK

**Keywords:** Snakebite, Systematic Review, Clinical Trials, Outcome Assessment, Treatment Outcome, Patient Reported Outcome Measures

## Abstract

Introduction:

A core outcome set (COS) is a minimal list of consensus outcomes that should be used in all intervention research in a specific domain. COS enhance the ability to undertake meaningful comparisons and to understand the benefits or harms of different treatments. A first step in developing a COS is to identify outcomes that have been used previously. We did this global systematic review to provide the foundation for development of a region-specific COS for snakebite envenomation.

Methods:

We searched 15 electronic databases, eight trial registries, and reference lists of included studies to identify reports of relevant trials, protocols, registry records and systematic reviews. We extracted verbatim data on outcomes, their definitions, measures, and time-points. Outcomes were classified as per an existing outcome taxonomy, and we identified unique outcomes based on similarities in the definition and measurement of the verbatim outcomes.

Results:

We included 107 records for 97 studies which met our inclusion criteria. These reported 538 outcomes, with a wide variety of outcome measures, definitions, and time points for measurement. We consolidated these into 88 unique outcomes, which we classified into core areas of mortality (1, 1.14 %), life impact (6, 6.82%), resource use (15, 17.05%), adverse events (7, 7.95%), physiological/clinical (51, 57.95%), and composite (8, 9.09%) outcomes. The types of outcomes varied by the type of intervention, and by geographic region. Only 15 of the 97 trials (17.04%) listed Patient Related Outcome Measures (PROMS).

Conclusion:

Trials evaluating interventions for snakebite demonstrate heterogeneity on outcomes and often omit important information related to outcome measurement (definitions, instruments, and time points). Developing high quality, region-specific COS for snakebite could inform the design of future trials and improve outcome reporting. Measurement of PROMS, resource use and life impact outcomes in trials on snakebite remains a gap.

## Background

Snakebite is a major public health problem in South Asia, Africa, and South America with an estimated 5.4 million people being bitten by snakes annually. It is estimated that snakebite causes up to 138,000 deaths worldwide each year, with three times as many people experiencing permanent disabilities. ^1^ In 2017, the World Health Organization (WHO) classified snakebite envenoming as a neglected tropical disease and this was followed by the launch of the WHO global strategy to reduce the mortality and morbidity by 50% by 2030. ^2^ One of the four key pillars of this strategy is to “ensure safe, effective treatment of snakebite”. ^2^ However, clinical practice guidelines, including those from the WHO, have been found to have quality issues, including in the use of evidence to inform recommendations for snakebite management. ^3^ These issues in guidelines is linked to the poor evidence base for interventions for snakebite management. We had previously found that systematic reviews on snakebite were of critically low quality. ^4^ Investment in treatments for snakebite, including in the identification of new therapies, has increased in recent years, and this trend is expected to continue. ^5^ Our earlier overview of systematic reviews on snakebite management has also highlighted the limitation of non-standardised measurement and reporting of outcomes. ^4^ This non standardisation of outcomes limits the ability of researchers, healthcare providers, decision makers, and patients to undertake meaningful comparisons and understand the potential benefits or harms of different treatment modalities. ^6^^,^^7^ Thus, there is an identified need for a core outcome set (COS) ^8^ for intervention research on snakebite management including trials and systematic reviews. A COS is a minimal list of consensus outcomes that should be used in all clinical trials and evidence synthesis in a specific area or setting of health or health care.

The objective of this study is to identify what outcomes have been used in intervention research on snakebite through a global systematic review of outcomes. This conduct of a robust and comprehensive systematic review of outcomes is an essential first step in the development of a COS. ^8^

## Methods

### Protocol, registration, and reporting

This systematic review is a part of a larger project to develop a COS for intervention research on snakebite in South Asia. The protocol for the entire project, including the current systematic review was registered *a priori* ( https://doi.org/10.17605/OSF.IO/PEKSJ). The COS development was registered *a priori* in the COMET database ( https://cometinitiative.org/Studies/Details/1849). A summary of the methods for this systematic review is provided below.

The PRISMA checklist for this report of the review is available in **Extended Dataset: Appendix 1** **.** ^9^

### Eligibility criteria

We included studies which met the following criteria:

• **Health condition/Population**: people with snakebite, irrespective of their sex/gender, species of snake, region, or any other factor.
• **Intervention**: any intervention regarding management of snakebite.
• **Comparators**: an active comparator or control group.
• **Outcomes**: the outcomes measured and reported, given the objective of identifying the full range of all outcomes.
• **Study Design**: we included studies with the following designs:
  o Randomised trials.
  o Non-randomised controlled trials.
  o Secondary analysis of randomised or non-randomised controlled trials.
  o Systematic reviews that included randomised or non-randomised controlled trials.
  We excluded systematic reviews which solely included non-trial designs. Protocols and trial registry records pertaining to the above were also included.
• **Other criteria**: there were no limits based on date of publication.

### Search strategy

We searched 15 electronic databases (PubMed, EMBASE, CINAHL, Cochrane Database of Systematic Reviews, ACP Journal Club, Database of Abstracts of Reviews of Effects, Cochrane Clinical Answers, Cochrane Central Register of Controlled Trials, Cochrane Methodology Register, Health Technology Assessment, NHS Economic Evaluation Database, Campbell Library, Epistemonikos, Scielo and Open dissertations) on 29 ^th^ October 2021, with no language restrictions. The search strategies for all databases are presented in **Extended Dataset: Appendix 2** **.** ^9^

We hand-searched nine trial registries (Australia New Zealand Trial Clinical Registry, Brazilian Clinical Trials Registry, Clinical Trial Registry of India, US trial registry ( clinicaltrials.gov), Iranian Registry of Clinical Trials, Thai Clinical Trials Registry, Peruvian Clinical Trial Registry, Sri Lanka Clinical Trials Registry, and WHO International Clinical Trial Registry Platform) in November 2021. We also screened the reference lists of included studies and contacted snakebite experts to identify additional eligible studies.

### Selection of articles

At least two reviewers (SB and DB or JT) independently screened the records retrieved based on titles and abstracts (where available) in the first phase and subsequently screened the full texts of potentially eligible studies. Disagreements, if any, were resolved by consensus between three reviewers (SB, DB, and JT).

### Data collection and management

We extracted data using a standardised data extraction form in REDCap (a secure web application for building and managing online surveys and databases) containing key information pertaining to participant details (number and demographics: age, sex/gender, country and time period of the study), details on the bite (species information), study design, intervention and comparator group, reported outcomes (together with their definitions, measurement instruments and timepoints). The outcomes from trials were supplemented by additional outcomes from systematic reviews. All details pertaining to outcomes were extracted verbatim as recommended by the COMET Handbook. ^8^

### Data synthesis

We analysed the verbatim information pertaining to reported outcomes. If we found multiple reports for the same study, we included these, but outcomes duplicated across these reports were only counted once. Outcomes which were not reported in the included trials, but which were defined or searched for in systematic reviews were also extracted verbatim. We classified the verbatim outcomes as per a taxonomy structure for outcomes in medical research ^10^ which has been validated for various health conditions. As such, we mapped outcomes areas (mortality, physiological or clinical, life impact, resource use and adverse events) and sub-domains within these. We had an additional domain for composite outcomes, recognising that their individual elements might not be categorised as “unique” under the other domains so that information on composite outcomes could be used in the next steps in the COS development. We consolidated the outcomes based on similarity of outcome measures and definitions to create a set of the unique outcomes that had been used in intervention studies on snakebite envenomation. We summarized the results using frequencies and percentages for these unique outcomes.

### Variation from published protocol

Our protocol envisaged inclusion of studies published from 1990 onwards. However, we searched for studies irrespective of date of publication and removed this time limit. We also searched electronic databases and trial registries that were not listed in our protocol to enhance comprehensiveness. On a post-hoc basis, we included secondary analysis of trials because some outcomes are not reported as a part of the main publication of a trial. This inclusive approach helped capture maximal evidence. We did not separate outcomes by different age-groups and special populations as originally planned because of the lack of studies. The decision to retain composite outcomes was post-hoc.

### Ethical approval

No ethical approval is required for this study because it is a systematic review of existing studies and does not include any human or animal participants.

## Results

### Study selection

**Figure 1.**
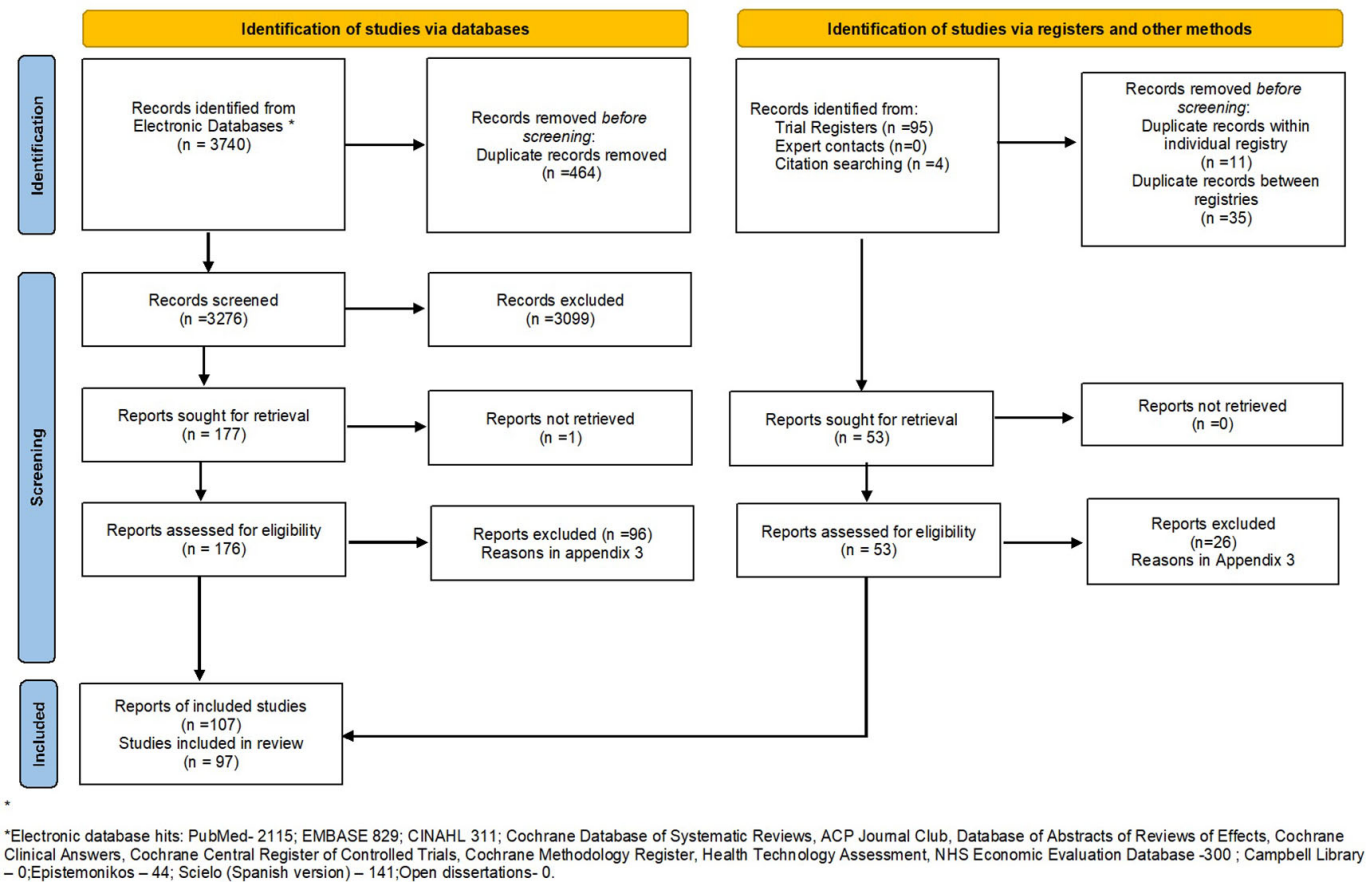
PRISMA flowchart showing selection of studies in the systematic review.

We found 3277 records in our search in electronic databases, 69 in trial registries and another two records through hand-searching relevant websites. After removing duplicates, obtaining, and evaluating full texts, 107 records from 97 studies met our eligibility criteria and are included in this review. A detailed PRISMA flowchart showing the inclusion of studies is presented in Figure 1.

Reasons for exclusion of records that were assessed at the full text level are shown in **Extended Dataset: Appendix 3** **.** ^9^

### Characteristic of included studies

We included nine systematic reviews, ^4^^,^^11^^–^^18^ and 88 trials and registry records. Out of the 88 trials and registry records, 84 are randomised trials, ^19^^–^^98^ and 4 are non-randomised controlled trials. ^99^^–^^102^ We found 10 post-hoc or secondary analysis of trials. ^103^^–^^112^ Out of the 84 randomised trials, there was one adaptive, ^68^ one factorial ^67^ and one cross-over trial. ^66^

The sample size ranged from eight to 1007 participants. Among the included trials, (26, 29.50%) were multicentric. ^19^^–^^23^^,^^26^^–^^28^^,^^34^^,^^41^^,^^49^^,^^50^^,^^55^^,^^64^^,^^66^^–^^68^^,^^70^^,^^74^^,^^81^^,^^85^^,^^89^^,^^90^^,^^99^^,^^113^^,^^114^ Three (3.41%) trials were exclusively on children. ^23^^,^^25^^,^^76^ Most of the studies (72, 81.8%) had only two arms of comparison. In 16 (18.2%) trials ^20^^,^^27^^,^^30^^,^^34^^,^^38^^,^^41^^,^^42^^,^^60^^,^^67^^,^^69^^,^^71^^,^^80^^–^^83^^,^^115^ with more than two comparison arms, the number of arms ranged from three to eight. A total of 49 (55.7%) trials ^19^^–^^56^^,^^77^^,^^78^^,^^80^^,^^82^^,^^85^^,^^88^^,^^93^^,^^98^^,^^101^^,^^102^ were restricted to participants with bites of a specific snake species/genus. Most of the trials started recruitment after the year 2000 (58, 65.91%).

A summary of the characteristics of the included studies is presented in Table 1 below.

**Table 1.** Characteristics of included studies.

Study Designs	• Randomised trials: 84 (and 10 post-hoc or secondary analysis from these) • Non-randomised controlled trials: 4 • Systematic reviews: 9		
Sample Size of trials	• Range is 80 to 1007 (median=80) • 0-50 participants: 24 (27.27%) • 51-100 participants: 27 (30.68%) • 101-200 participants: 26 (29.54%) • >200 participants: 11 (12.50%)		
Decadal Periods (based on start year of recruitment)	• ≤1990:14 • 1991-2000: 13 • 2001-2010: 27 • 2011-2020: 27 • ≥2021: 4 • Unclear/not reported: 3		
Number of centres in trials	• Multicentre: 26 (29.5%) • Single centre: 62 (70.5%)		
Snake species in trials	• Restricted to bites of specific snake species/genus: 49 (55.7%) • Not restricted to bites of specific snake species/genus: 39 (44.3%)		
Country of conduct of trials	**Country**	**Number of trials**	**%**
	Australia	2	2.27
	Brazil	9	10.23
	China	4	4.54
	Colombia	6	6.82
	Ecuador	2	2.27
	India	17	19.32
	Iran	2	2.27
	Malaysia	1	1.14
	Mexico	2	2.27
	Myanmar	3	3.41
	Nepal	1	1.14
	Central African Republic	1	1.14
	Nigeria	6	6.82
	Pakistan	1	1.14
	Papua New Guinea	1	1.14
	Philippines	2	2.27
	Sri Lanka	15	17.04
	Thailand	5	5.68
	United States	7	7.95
	Vietnam	1	1.14

### Synthesis of outcomes

We extracted verbatim data for 538 outcomes and categorised them into the following core areas: death/mortality (26, 4.83%), life impact (19, 3.53%), resource use (96, 17.84%), adverse events (80, 14.87%), physiological/clinical (288, 53.53%), and composite outcome (29, 5.39%). The proportionate frequency of outcomes by domain and sub-domains of physiological/clinical outcomes varied both by the type of intervention being evaluated and by geographic region ( **Extended Dataset: appendix 4** ^9^). Trials from South Asia seldom measured life impact outcomes (0.47% of trials in contrast to 17.05% of North American trials which reported on life impact outcomes), but they frequently (29.28%) reported resource use outcomes. The focus of trials from South America, Southeast Asia and the rest of Asia is overwhelmingly in physiological/clinical outcomes. Blood and lymphatic system outcomes were proportionately much higher in African trials (64.00%) compared to South Asian (35.63%) trials. South Asian trials measured renal outcomes more (in 18.39% trials) compared to trials in other regions. Reporting of respiratory outcomes were uncommon except in Australia and Papua New Guinea (11.11%) trials.

Only 48 trials (54.54%) specifically identified their primary outcomes, out of which 10 (11.37%) ^47^^,^^60^^–^^62^^,^^67^^,^^77^^,^^84^^,^^89^^,^^97^^,^^98^ had adverse events or effects as a primary outcome.

We consolidated the verbatim outcomes into 88 unique outcomes which we categorised as: mortality (1, 1.14%), life impact (6, 6.82%), resource use (15, 17.05%), adverse event (7, 7.95%), physiological/clinical (51, 57.95%), and composite (8, 9.09%).

The long list of the unique outcomes with summary information on their measurement and definitions is provided in Table 2 and discussed below.

**Table 2.** List of unique outcomes identified in systematic review and summary information.

Outcome area	Outcome summary
**Mortality**	Death was measured as all-cause mortality, survival, or cause-specific mortality
**Life Impact Outcome**	1. Functional life impact: Patient Specific Functional Scale, and the physical function domain of the SF-36 questionnaire 2. Disability: Sheehan Disability Inventory and American Medical Association (AMA) disability rating score. 3. Quality of life: Patient's Global Impression of Change Scale, Clinical Global Impression - Improvement (CGI-I), and Patient-reported outcome measurement information system physical function-10 score (PROMIS PF-10). 4. Time to functional recovery: defined as time to full functional status recovery as measured by the Patient-Specific Functional Scale, or complete resolution of swelling and ability to run and jump (for lower extremity bites) or equal hand-grip (for upper extremity bites). 5. Lower extremity function: Scores on Lower Extremity Functional Scale, and walking speed. 6. Upper extremity function: Scores on the Disorders of the Arm, Shoulder, and Hand (DASH) and grip strength through a dynamometer.
**Resource use outcome**	**Hospital** 1. Duration of hospital stay: no clear criterion for discharge except in one study. 2. Duration of Intensive Care Unit (ICU) stay: no clear criterion.
	**Need for further intervention** 1. Requirement of a blood product (unspecified or any). 2. Requirement of FFP (fresh frozen plasma). 3. Requirement of PRBC (packed red blood cell). 4. Requirement of platelets. 5. Requirement of cryoprecipitate. 6. Requirement of mechanical ventilation. 7. Requirement for non-invasive ventilation or reintubation (Post- extubation). 8. Requirement of antibiotic. 9. Requirement of analgesic. 10. Requirement of dialysis/renal replacement therapy. 11. Requirement of antivenom.
	**Economic** 1. Cost of antivenom (average compared). 2. Any cost-related outcome.
**Adverse event/effect**	1. Adverse event unclassified: proportion and time from antivenom infusion to reaction with or without classification of severity or frequency/proportion of treatment emergent adverse event. 2. Anaphylaxis: incidence and time from antivenom infusion to anaphylaxis with or without classification (Brown 2004 criterion) of severity. 3. Anaphylactoid syndrome: incidence of anaphylactoid syndrome, pyrogenic reaction alone and urticaria alone. 4. Early antivenom reaction (EAR): incidence and time from antivenom infusion to EAR. 5. Late antivenom reaction: incidence. 6. Adverse events specific to FFP: incidence. 7. Capillary leak syndrome: incidence.
**Physiological/Clinical**	**Eye** 1. Conjunctival oedema.
	**Cardiac** 1. Cardiac rhythm abnormalities. 2. Hypotension. 3. Shock.
	**Psychiatric** 1. Anxiety: Hopkins somatic symptoms checklist. 2. Depression: modified Sinhala version of the Beck depression inventory. 3. Post-Traumatic Stress Disorder: Post-traumatic Stress Symptom Scale-Self Report Scale. 4. Suicidal ideation and behaviour: Columbia-Suicide Severity Rating.
	**Respiratory, thoracic and mediastinal** 1. Respiratory distress: measured as airway obstruction, respiratory failure, and acute respiratory distress syndrome; no specific definition reported. 2. Negative inspiratory pressure: standard methods. 3. Forced vital capacity: standard methods.
	**Neurological** 1. Paralysis: proportion or duration; assessed clinically, no clear definition. 2. Ophthalmoplegia/Ptosis: days for resolution of ptosis/ophthalmoplegia, endurance of upward gaze, and proportion of the iris uncovered. 3. Anosmia: as reported by patient. 4. Motor strength: no clear definition. 5. Neurotoxicity overall: incidence/frequency and time to complete resolution of all neuroparalytic features.
	**Injury/Poisoning** 1. Venom concentration: standard methods. 2. Anti-venom concentration: standard methods. 3. Varisyllabic-methyl levels: standard methods.
	**Immunological** 1. Immunogenicity profile: standard methods. 2. profile of antibodies: standard methods. 3. COVIP-Plus induced sera: standard methods.
	**General** 1. Pain: intensity measured by Visual Analogue Scale, time for complete resolution of the local pain with or without induration. 2. Non-specific systemic symptoms: no definition provided.
	**Musculoskeletal** 1. Myotoxicity as an outcome was measured clinically, levels of creatine kinase, and levels of lactate dehydrogenase, creatine phosphokinase, metalloproteinases.
	**Skin and subcutaneous** 1. Necrosis: assessed clinically, no clear definition. 2. Blistering: assessed clinically, no clear definition. 3. Oedema: measured as circumference difference between the affected limb and the normal limb; circumference measurements of the affected limb alone; remission time of limb swelling; cessation of local swelling progression; time to swelling resolution; oedema progression; measurement of decrease of oedema-scaled dish. 4. Swelling: measured based on the number of segments affected (extent) and increase in circumference of the bitten limb (intensity); proximal length of swelling from bite site; criteria developed by Warell et al 1977; criteria based on physical appearance of swelling; swelling is confirmed to bitten segment or crosses 1 or 2 joints; and % increase in volume compared to contralateral (non-envenomated) limb. 5. Wound cosmesis: measured by any validated cosmesis score. 6. Any other wound related outcome, including but not limited to cosmesis.
	**Infection, Infestation, and Inflammation** 1. Abscess. 2. Blister. 3. Cellulitis. 4. Inflammatory markers. 5. Pneumonia. 6. Ventilator associated pneumonia. 7. Wound infection.
	**Kidney and Urinary Outcomes** 1. Blood urea nitrogen (BUN) and creatinine levels measures in serum. 2. Acute Kidney Injury defined as per Risk, Injury, Failure, Loss of kidney function, and End-stage kidney disease (RIFLE) Criteria, Kidney Disease Improving Global Outcomes (KDIGO) Criteria, measurement of surrogate markers (Neutrophil gelatinase-associated lipocalin, beta2-microglobulin, Kidney Injury Molecule-1, serum creatinine), estimated glomerular filtration rate and oliguria. 3. Abnormalities in urine: proteinuria or haematuria. 4. Chronic kidney disease: no definition provided. 5. Renal angle tenderness: no definition provided. 6. Ferryl-haem derivatives: detected in urine sample.
	**Blood and lymphatic system** 1. Blood coagulability -by 20 min whole blood clotting test (WBCT20)/Lee -White method, or standard laboratory measures of international normalized ratio (INR), bleeding time (BT), clotting time (CT), Prothrombin Time (PT), aPTT (activated partial thromboplastin time). 2. Platelet count- standard laboratory measures. 3. Clotting Factors- Clotting factor panel or specific factors like fibrinogen, Factor V, VII, VIII, Fibrinogen degradation products/D-dimer. 4. Bleeding – defined clinically using various criterion. 5. Clot Quality- measures as per a method developed by Reid 6. Other Haematological parameters – complete blood count, packed cell volume. 7. Lymphadenopathy/lymphadenitis – no clear clinical criteria provided.
**Composite Outcome**	1. Clinical recovery as a composite outcome: seven unique definitions were used. 2. Complications as a composite outcome: four different definitions were used or not clearly reported. 3. Envenoming manifestations: measured compositely as improvement in signs and symptoms of envenoming (systematic alone or together with local). 4. Snakebite Severity Score (SSS): either the complete SSS or the pulmonary, cardiovascular, hematologic symptoms, and nervous system sub scores of the SSS, and as defined in the US FDA-approved information for Crotaline Polyvalent Immune Fab antivenom (FabAV) prescription. 5. Haemolysis: measured using haemolysis markers (visual haemolysis score level and abnormal lactate dehydrogenase - LDH levels). 6. Local Inflammation: Reduction in local inflammatory manifestations such as pain, oedema, and temperature (flushing). 7. Prognosis: no clear definition. 8. Treatment failure: measured as a composite outcome based on clinical features.

**Death/mortality outcomes**

We found one unique outcome (1.14%) for mortality from 26 verbatim outcomes (4.83%) in 26 trials and five systematic reviews. Time points at which death was measured were until discharge from hospital, 28 days from discharge, 60 days from recruitment/intervention and 90 days from bite.

More detailed information tabulating this unique outcome, together with measures, definitions and time-points, is reported in **Extended Dataset: Appendix 5**. ^9^

**Life impact outcomes**

We found six unique outcomes (6.82%) from 19(3.53%) verbatim outcomes from six trials and one systematic review. No trials or systematic reviews measured any life impact outcome related to social functioning, emotional functioning/well-being, cognitive functioning, perceived health status, compliance (including withdrawal from treatment), delivery of care or personal circumstances.

A clear definition with clear details on the outcome measurement instruments used was provided for all but one verbatim life impact outcome. Disability outcomes were measured long term (six months from discharge and 12 months from intervention). Serial measurement from baseline through 28 days (from bite) was common. More detailed information about these unique outcomes, together with their measures, definitions and time-points for measurements is provided in **Extended Dataset: Appendix 5**. ^9^

**Resource use outcomes**

We found 15 unique (17.05%) resource use outcomes from 96 verbatim (17.84%) outcomes: two (2.27%) hospital use outcomes from 30 verbatim (5.58%) outcomes in 25 trials; 11 (12.5%) outcomes related to the need for further intervention from 65 verbatim (12.08%) outcomes in 25 trials, and two (2.27%) economic outcomes from two verbatim outcomes (0.37%) from one trial and one systematic review. No trials or systematic reviews reported on outcomes of societal or carer economic burden.

For almost all the resource use outcomes, the specific clinical criterion associated with the outcome was not reported. For example, none of the outcomes relating to the need for further intervention reported the specific clinical criteria that would lead to further intervention. More detailed information about these unique outcomes together with their measures, definitions and time-points for measurement is provided in **Extended Dataset: Appendix 5**. ^9^

**Adverse effect/events outcomes**

Our synthesis led to seven (7.95%) unique adverse event outcomes from a total of 80 (14.87%) verbatim outcomes from 52 trials and six systematic reviews. There was substantial heterogeneity in the definitions used for these outcomes. Adverse effects and events were almost always measured in the acute setting with no long-term measurement of these outcomes. More detailed information about these unique outcomes together with their measures, definitions and time-points for measurement is provided in **Extended Dataset: Appendix 6**. ^9^

**Physiological or clinical outcomes**

The 288 (53.53%) verbatim physiological/clinical outcomes from trials were consolidated into 51(57.95%) unique physiological/clinical outcomes and were classified as per the taxonomy into the following:

• **Eye**: one unique (1.14%) outcome from one (0.19%) verbatim outcome from one trial. Outcomes were assessed clinically with no clear criteria reported in the trials.
• **Cardiac**: three (3.41%) unique outcomes from seven (1.30%) verbatim outcomes from six trials. Outcomes were assessed clinically with no clear criteria reported in the trials.
• **Psychiatric**: four (4.55%) unique outcomes from four (0.74%) verbatim outcomes from two trials and one systematic review. psychiatric outcomes were measured with validated instruments and had good reporting of time points.
• **Respiratory, thoracic, and mediastinal**: three (3.41%) unique outcomes from four (0.74%) verbatim outcomes from three trials. Two of the outcomes are related to standard spirometry tests while the other was assessed clinically with no clear definition provided.
• **Nervous system:** five (5.68%) unique outcomes from 16(2.97%) verbatim outcomes from 13 trials and one systematic review. Many of the outcomes were measured clinically with no specific criteria mentioned.
• **Injury and poisoning outcomes**: three (3.41%) unique outcomes from 28(5.20%) verbatim outcomes from 20 trials. All the outcomes were laboratory measured.
• **Immune system**: three (3.41%) unique outcomes from three verbatim outcomes from one trial. All were laboratory measures.
• **General:** two (2.27%) unique outcomes from 16 (2.97%) verbatim outcomes from 14 trials. A standardised tool was used for one outcome and a clear definition was not provided for the other. Time points were not clear for both.
• **Musculoskeletal and connective tissue**: one (1.14%) unique outcome from eight (1.49%) verbatim outcomes from seven trials. Clear definitions were provided for the outcome measures and pertained to use of standard laboratory tests.
• **Skin and subcutaneous tissue outcomes**: six (6.82%) unique outcomes from 41 (7.62%) verbatim outcomes from 29 trials and one systematic review. Outcomes were assessed clinically with no clear criteria or time points reported for many outcome measures.
• **Renal and urinary:** six (6.82%) unique outcomes from 26(4.83%) verbatim outcomes from 14 trials and one systematic review. Outcome definitions were clearly reported (except for one outcome) and validated criteria or standard laboratory methods were used.
• **Infection, infestation, and inflammation**: seven (7.95%) unique outcomes from 21(3.90%) verbatim outcomes from 15 trials and 1 systematic review. There was substantial heterogeneity in outcome measures and definition with reporting being poor when clinical assessment was the basis of outcome measurement.
• **Blood and lymphatic system:** seven (7.95%) unique outcomes from 122(22.68%) verbatim outcomes from 49 trials and five systematic reviews. Laboratory tests were the basis of six of these outcomes with clinical assessment being the basis of outcome measurement in the other three.

No trial or systematic review measured endocrine outcomes, ear and labyrinth outcomes, gastrointestinal outcomes, hepatobiliary outcomes, puerperium, and perinatal outcomes, or vascular outcomes. We considered the following taxonomy sub-categories to be not relevant to the snakebite: familial, and genetic outcomes, metabolism and nutrition outcomes, outcomes relating to neoplasms: benign, malignant, and unspecified (including cysts and polyps), reproductive system and breast outcomes.

In general, reporting of time points was poor in many trials. More detailed information about these unique outcomes together with their measures, definitions and time-points for measurement is provided in **Extended Dataset: Appendix 7**. ^9^

**Composite outcomes**

Our synthesis led to eight (9.09%) unique composite outcomes from 29(5.39%) verbatim outcomes from 21 trials and one verbatim outcome from two systematic reviews. There was substantial heterogeneity in outcome definitions as well as in the time points for measurement.

More detailed information on these unique outcomes together with measures, definitions and time-points for measurement is provided in **Extended Dataset: Appendix 8**. ^9^

### Patient reported outcome measures

Only 15 trials ^22^^,^^66^^,^^78^^,^^82^^,^^88^^,^^90^^,^^116^ included Patient Reported Outcome Measures (PROMs). The PROMs used in snakebite trials (with related citations on the measurement tools, where relevant) is presented in Table 3.

**Table 3.** Patient Reported Outcome Measures Reported in Snakebite Trials *.

• Pain related o Visual Analog Scale ^128^ o Numeric Pain Rating Scale ^128^ • Physical Function/disability related o Patient Specific Functional Scale ^110^^,^^129^ o Physical function domain of the SF-36 questionnaire ^130^ o Sheehan Disability Inventory ^131^^,^^132^ o American Medical Association disability rating score ^133^ o Patient's Global Impression of Change Scale ^134^ o Patient-reported outcome measurement information system physical function-10 ^135^ o Lower Extremity Functional Scale ^136^ o Disorders of the Arm, Shoulder, and Hand ^137^ o Anosmia-as reported by patient • Mental Health related o Hopkins somatic symptoms checklist ^131^^,^^138^ o Beck depression inventory ^131^^,^^139^(modified Sinhala version) o Post-traumatic Stress Symptom Scale-Self Report Scale ^131^^,^^140^ o Columbia-Suicide Severity Rating ^141^

* Details on PROMS is within different Extended Dataset appendices.

## Discussion

This systematic review of outcomes captured a total of 538 verbatim outcomes, which were consolidated into 88 unique outcomes, which had a wide variety of measures, definitions, and time points for their measurement. Outcome definitions and time points were infrequently or poorly reported. Outcomes related to resource use and life impact were included in few trials, with no trials using outcome related, societal or carer burden, social functioning, emotional functioning or well-being, cognitive functioning, perceived health status, compliance, and delivery of care or personal circumstances. The only trial which captured economic outcomes reported the average cost of antivenom without any comprehensive calculation of other direct or indirect costs. No trials had outcomes related to pregnancy. We also found that the primary outcome was explicitly stated in only a few of the trials. Outcome types (by the taxonomy domains) varied for both different geographic regions and different types of intervention being evaluated.

Heterogeneity in outcomes, their measures, definitions, and time points prevents comparison of effectiveness data, thus limiting the usefulness of trials and reviews to clinical practice. ^8^ The major reason for evidence synthesis specialists not being able to conduct meta-analyses is heterogeneity in the ways in which outcomes are reported and measured. ^8^^,^^117^ This systematic review has shown that this is likely to be a major problem for snakebite research. Therefore, if high-quality trials are to be done to develop a robust evidence base for the management of snakebite, ^4^ this needs to be preceded by the development of COS for use in intervention research.

The variation in outcomes (by taxonomically domains) across geographic regions and types of intervention indicates the need for development of COS with a focus on specific regions. This is to be expected because the clinical features of envenomation and the consequent choice of type of intervention largely depends on the type of snake and this largely depends on the geographic region. As such, this review has confirmed the appropriateness of our intention to develop a COS with a specific South Asia focus. This is also in keeping with the WHO’s work to develop targeted therapeutic profiles for snakebite envenomation therapies on a geographic region basis. ^118^ The scarce use of PROMs, resource use and life impact outcomes in snakebite research to date is a gap that needs to be filled. These outcomes have high relevance for, for example, patients, clinicians, and hospital administrators. Our systematic review identified several outcome measurements instruments, but their measurement properties (structural validity, internal consistency, cross-cultural validity, and reliability) and the feasibility of using them is unknown. We will address this in future steps of our COS development, which will not only involve public health professionals, social workers, health economists, but also patients and caregivers of people with snakebite. This would enhance the clinical and public health relevance of the COS. To achieve this, the inventory of outcomes from this systematic review will be supplemented by additional outcomes identified by stakeholders participating in the consensus development process in the next steps of COS development. ^8^

There is also a need for better reporting of outcome definitions (including outcome measurement instruments) and time points of measurement in clinical trials for snakebite. While the uptake of COS in future trials ^119^ will address this issue to some extent, there is also a need for funders, trialists, and journal editors to take this into account through the lifecycle of a trial, from its design stage to publication. The lack of specific mention of primary outcomes also needs to be addressed. ^120^ Trialists should also ensure that their outcome measurement instruments are valid for their own setting or use ones which are validated in their geographic region, such that their cross-cultural validity and feasibility is enhanced. ^121^ Improvements in trial outcome transparency and reporting should also arise from the forthcoming Outcome extensions for the Standard Protocol Items: Recommendations for Intervention Trials (SPIRIT) and CONsolidated Standards of Reporting Trials (CONSORT) statements. ^122^ Beyond improvements in how outcomes are used in trials, there is also a need to conduct more trials in children, since we identified only three trials exclusively on children.

Strengths of this review include that we searched 15 electronic databases and eight trial registries to comprehensively capture outcomes from as many studies as possible, not only including those trials which have completed but also including trials that are not published, not completed, or were terminated early. We have used standard evidence synthesis methods to maintain quality and used a validated taxonomy to classify outcomes. In keeping with guidance on the use of the taxonomy, this standardised outcome classification system allowed us to remove “inconsistencies due to ambiguity and variation in how outcomes are described across different studies”. ^10^ We acknowledge the limitation that, although we were able to successfully manage records in English and Spanish, we were unable to extract information from five records ^123^^–^^127^ that were available in Portuguese and Chinese.

## Conclusion

We have shown that trials evaluating therapies for snakebite envenoming have heterogeneity of outcomes and often omitted key information related to their measurement. Developing high quality region-specific COS for snakebite would facilitate improvements in the design and reporting of future trials and thereby strengthen their ability to have a positive impact on policy, practice, patient care and overall health. Particular attention also needs to be paid to improve the reporting of outcomes, and to include PROMs, resource use and life impact outcomes in trials on snakebite.

## Data availability

### Underlying data

Figshare. Outcomes in intervention research on snakebite envenomation: a systematic review. DOI: https://doi.org/10.6084/m9.figshare.19777921.v1

This project contains the following underlying data in the extended dataset:

• Data file Appendix 1. PRISMA checklist
• Data file Appendix 2. Search strategies of all databases
• Data file Appendix 3. Reasons for exclusion at full text level
• Data file Appendix 4. Outcome core categories by region and intervention
• Data file Appendix 5. Detailed data on outcomes in categories of death, life impact and resource use
• Data file Appendix 6. Detailed data on outcomes in categories of adverse event
• Data file Appendix 7. Detailed data on outcomes in categories of physiological/clinical
• Data file Appendix 8. Detailed data on composite outcomes

Data are available under the terms of the Creative Commons Attribution 4.0 International license (CC-BY 4.0).
