# Supplementary material for: Outcomes in intervention research on snakebite envenomation: a systematic review
Source: F1000Res. 2022 Jun 8;11:628. [Version 1] doi: 10.12688/f1000research.122116.1 (PMC9579743; doi:10.12688/f1000research.122116.1)
Supplement: Peer review history

# Peer review: Reviewer response for version 1

**DOI:** 10.5256/f1000research.134062.r153142

**Published:** 2022-10-18

**Authors:** Julien Potet

**Affiliations:** 1 Médecins Sans Frontières, Paris, France

The authors have conducted a thorough review of published clinical trial studies and have meticulously listed all the outcomes used in these studies. This review demonstrates heterogeneity on outcomes. This review will be helpful to support efforts to determine a minimal list of consensus outcomes (aka a Core Outcome Set) that should be used in forthcoming trials of interventions against snakebite. 

 My only criticism is that authors have omitted to mention and discuss a very similar review of existing clinical outcomes for snakebite trials that was published in PLoS-NTD in 2021 (Abouyannis *et al.* 2021) ^1^. It would be interesting to compare the results of the authors' review with the results of this other PLoS-NTD review.

 Apart from that, I want to commend the authors for this impressive work!

Is the work clearly and accurately presented and does it cite the current literature?

Partly

If applicable, is the statistical analysis and its interpretation appropriate?

Yes

Are all the source data underlying the results available to ensure full reproducibility?

Yes

Is the study design appropriate and is the work technically sound?

Yes

Are the conclusions drawn adequately supported by the results?

Yes

Are sufficient details of methods and analysis provided to allow replication by others?

Yes

Reviewer Expertise:

Antivenom development, antivenom access

I confirm that I have read this submission and believe that I have an appropriate level of expertise to confirm that it is of an acceptable scientific standard.

## References

[1] : Clinical outcomes and outcome measurement tools reported in randomised controlled trials of treatment for snakebite envenoming: A systematic review. *PLoS Negl Trop Dis* .15(8) : 10.1371/journal.pntd.0009589 e0009589 10.1371/journal.pntd.0009589 34339410PMC8360524

# Peer review: Reviewer response for version 1

**DOI:** 10.5256/f1000research.134062.r144412

**Published:** 2022-07-20

**Authors:** Harry Williams

**Affiliations:** 1 Toxiven Biotech Private Limited, Coimbatore, Tamil Nadu, India

Generally this is a superb review. The authors have carried out an incredibly detailed systematic review and this article meets all the criteria f1000 set for acceptable publications and has hopefully paved the way for their goal of a South Asian Core Outcome Set to be developed. The article is well written with very impressive tables of results and as a precursor to the eventual COS is exemplary for the methodology to be used in this process. Overall Bhaumik et al. have done a great job at reviewing the vast and varied snakebite literature and I look forward to reading the eventual COS. They have reviewed the literature to identify the full range of outcomes associated with snakebite envenomation. They identify the need to homogenise outcome reports so as to easily compare cases and act fast following the admission of a previously documented outcome. This would enable future clinical trials to be designed more methodically.

 Major Points:

1. At no point in their review do they mention any of the snake species involved or any means for diagnosing the bites and ensuring they are indeed snake bites. 
2. None of the toxins found within snake venoms are mentioned or attributed to any of the effects seen in these cases. I understand this was not the purpose of the review but it would have added greatly to my personal enjoyment if there was at least some level of discussion surrounding each of the unique outcomes regarding the snakes or toxins to blame.

Is the work clearly and accurately presented and does it cite the current literature?

Yes

If applicable, is the statistical analysis and its interpretation appropriate?

Not applicable

Are all the source data underlying the results available to ensure full reproducibility?

Yes

Is the study design appropriate and is the work technically sound?

Yes

Are the conclusions drawn adequately supported by the results?

Yes

Are sufficient details of methods and analysis provided to allow replication by others?

Yes

Reviewer Expertise:

Snakebite research, human-wildlife conflict, toxicology

I confirm that I have read this submission and believe that I have an appropriate level of expertise to confirm that it is of an acceptable scientific standard.
